# Love and affectionate touch toward romantic partners all over the world

**DOI:** 10.1038/s41598-023-31502-1

**Published:** 2023-04-04

**Authors:** Agnieszka Sorokowska, Marta Kowal, Supreet Saluja, Toivo Aavik, Charlotte Alm, Afifa Anjum, Kelly Asao, Carlota Batres, Aicha Bensafia, Boris Bizumic, Mahmoud Boussena, David M. Buss, Marina Butovskaya, Seda Can, Antonin Carrier, Hakan Cetinkaya, Daniel Conroy-Beam, Rosa María Cueto, Marcin Czub, Seda Dural, Agustín Espinosa, Carla Sofia Esteves, Tomasz Frackowiak, Jorge Contreras-Garduño, Farida Guemaz, Ivana Hromatko, Herak Iskra, Feng Jiang, Konstantinos Kafetsios, Tina Kavcic, Nicolas Kervyn, Nils C. Köbis, Aleksandra Kostić, András Láng, Torun Lindholm, Zoi Manesi, Norbert Meskó, Girishwar Misra, Conal Monaghan, Jean Carlos Natividade, George Nizharadze, Elisabeth Oberzaucher, Anna Oleszkiewicz, Ariela Francesca Pagani, Vilmante Pakalniskiene, Miriam Parise, Marija Pejičić, Annette Pisanski, Kasia Pisanski, Camelia Popa, Pavol Prokop, Ruta Sargautyte, Shivantika Sharad, Franco Simonetti, Piotr Sorokowski, Michal Mikolaj Stefanczyk, Anna Szagdaj, Meri Tadinac, Karina Ugalde González, Olga Uhryn, Christin-Melanie Vauclair, Gyesook Yoo, Maja Zupančič, Ilona Croy

**Affiliations:** 1 Institute of Psychology, University of Wroclaw, Ul. Dawida 1, 50-527 Wroclaw, Poland; 2 Macquarie University, Sydney, Australia; 3 University of Tartu, Tartu, Estonia; 4 Stockholm University, Stockholm, Sweden; 5 University of the Punjab, Lahore, Pakistan; 6 Westminster College, Salt Lake City, USA; 7 Franklin and Marshall College, Lancaster, USA; 8 University of Algiers, Algiers, Algeria; 9 Australian National University, Canberra, Australia; 10 University Setif2, Setif, Algeria; 11 University of Texas at Austin, Austin, USA; 12 Russian Academy of Sciences, Moscow, Russia; 13 Izmir University of Economics, Izmir, Turkey; 14 Université catholique de Louvain, Louvain-la-Neuve, Belgium; 15 Yasar University, Izmir, Turkey; 16 University of California, Santa Barbara, Santa Barbara, USA; 17 Pontificia Universidad Católica del Perú, Lima, Peru; 18 Universidade Católica Portuguesa, Lisboa, Portugal; 19 Escuela Nacional de Estudios Superiores, UNAM, Morelia, Mexico; 20 University of Zagreb, Zagreb, Croatia; 21 University of Greenwich, London, UK; 22 Department of Psychology, University of Crete, Rethymno, Crete Greece; 23 University of Ljubljana, Ljubljana, Slovenia; 24 Max Planck Institute for Human Development, Berlin, Germany; 25 University of Niš, Niš, Serbia; 26 University of Pécs, Pécs, Hungary; 27 Vrije Universiteit Amsterdam, Amsterdam, The Netherlands; 28 University of Delhi, Delhi, India; 29 Pontifical Catholic University of Rio de Janeiro, Rio de Janeiro, Brazil; 30 Free University of Tbilisi, Tbilisi, Georgia; 31 University of Vienna, Vienna, Austria; 32 University of Urbino, Urbino, Italy; 33 Vilnius University, Vilnius, Lithuania; 34 Università Cattolica del Sacro Cuore, Milan, Italy; 35 University of Alberta, Edmonton, Canada; 36 CNRS, University of Lyon 2, Lyon, France; 37 Romanian Academy, Bucharest, Romania; 38 Comenius University, Bratislava, Slovakia; 39 Universidad Catolica de Chile, Santiago, Chile; 40 Wroclaw University of Science and Technology, Wroclaw, Poland; 41 Universidad Latina de Costa Rica, San José, USA; 42 Lviv State University of Internal Affairs, Lviv, Costa Rica; 43 Instituto Universitário de Lisboa (ISCTE-IUL), Lisboa, Portugal; 44 Kyung Hee University, Seoul, South Korea; 45 Institute of Psychology, University of Jena, Jena, Germany; 46 Smell & Taste Clinic, Department of Otorhinolaryngology, Faculty of Medicine Carl Gustav Carus, Technische Universität Dresden, Dresden, Germany; 47 IDN Being Human Lab, University of Wrocław, Wrocław, Poland; 48 German Center for Mental Health, Halle-Jena-Magdeburg, Germany

**Keywords:** Neuroscience, Physiology, Psychology

## Abstract

Touch is the primary way people communicate intimacy in romantic relationships, and affectionate touch behaviors such as stroking, hugging and kissing are universally observed in partnerships all over the world. Here, we explored the association of love and affectionate touch behaviors in romantic partnerships in two studies comprising 7880 participants. In the first study, we used a cross-cultural survey conducted in 37 countries to test whether love was universally associated with affectionate touch behaviors. In the second study, using a more fine-tuned touch behavior scale, we tested whether the frequency of affectionate touch behaviors was related to love in romantic partnerships. As hypothesized, love was significantly and positively associated with affectionate touch behaviors in both studies and this result was replicated regardless of the inclusion of potentially relevant factors as controls. Altogether, our data strongly suggest that affectionate touch is a relatively stable characteristic of human romantic relationships that is robustly and reliably related to the degree of reported love between partners.

## Introduction

Touch is the primary way people communicate intimacy in romantic relationships^1^. Partners are touched significantly more often than are other people^2^ and individuals in a romantic relationship report significantly more intimate touch than do single people^3^. Even imagining a partner’s touch can evoke pleasant and erotic sensations^4^, and romantic partners are also typically accepted to touch a higher proportion of the body as compared to strangers or friends. For example, most people feel comfortable when they are touched in the abdomen and thighs by their partner, but not by other people^5^. Additionally, the types of affectionate touch performed in partnerships are more heterogeneous than in other social interactions^6^. A stroke, for instance, is performed with a particularly low velocity^7^ when it is directed to a romantic partner. In line with this, a recent cross-cultural examination showed that despite considerable intercultural differences, affectionate touch behaviors such as an embrace, caress, kiss, and hug, were universally present in partnerships all over the world^6^.

The particular tendency to use affectionate touch in a romantic relationship comes as no surprise given the adverse consequences of touch deprivation that are in stark contrast to many benefits of affectionate touch presence in close relationships. Touch deprivation relates to depression, anxiety and somatization^2^, while more partner-touch predicts better psychological well-being, also in a long-term perspective^8^. Furthermore, interpersonal touch can provide valuable support in difficult situations, as it contributes to a lower stress response^9^ through reducing heart rate and blood pressure^10–15^, as well as by decreasing cortisol production^10^. Touch can also alleviate pain through its effects on μ-opioids^16^ and serotonin levels^17^. Nonetheless, it should not be forgotten that touch may not be that positive under certain circumstances. Some people dislike touch (i.e., they are touch avoidant^18^) or react to interpersonal touch in a negative manner^19^. The negative response to touch may also be driven by factors related to the interaction partner (e.g., low familiarity^20^, or a disgust-evoking disease^21^).

The significant implications and consequences of affectionate touch may be interpreted in the context of Affection Exchange Theory (AET^22^). According to this theory, affectionate communication is crucial for “promoting the establishment and maintenance of significant human pair bonds’’^22^ (p. 165). Accordingly, as mentioned before, expressions of affection are particularly likely in couples^2,3,6^ and can be predictive of a romantic relationships’ quality. Individuals of higher relationship commitment report communicating affection (including displays of physical affection) toward their partners^23^. The degree of physical affection further correlates positively with relationship and partner satisfaction^24^, and negatively with attachment insecurity^25^. However, the affectionate communication referred to in most studies typically comprises several types of behaviors and verbal displays of affection [e.g., hugging was the only behavior explicitly related to touch among several affection communication components analyzed in Horan and Booth-Butterfield's study^23^]. In one of a few studies focused directly on touch in romantic partnerships, relationship quality was found to be positively associated with desire for touch, while attachment avoidance was associated with lower overall desire for touch^26^. Touch was further found to be a significant mediator between attachment patterns and relationship well-being^27^. Despite these promising findings and the obvious value of touch in close interpersonal relationships, scientific knowledge on affectionate touch in romantic relationships still remains rather sparse.

There also appears to be little research concerning psychological factors that determine the use of affectionate touch in couples. For example, it is logical to predict that loving partners would use more touch in their relationships, enriching communication, and enjoying the benefits typically associated with affectionate touch. Touch, at the same time, could promote love between partners, in line with a study showing that one’s own and one’s partner’s approach motives for touch predict greater daily relationship well-being^27^. This hypothesis finds support in an older study by Dainton, Stafford and Canary^28^ who showed that physical affection (including touch behaviors) performed by a romantic partner as well as satisfaction with physical affection displays were positively related with self-assessed love level. However, surprisingly, apart from this study little can be said on a direct relationship between affectionate touch and love—one of the most important components of human romantic relationships.

A prominent Triangular Theory of Love presents love as a construct consisting of three components, namely passion, intimacy, and commitment^29^. Passion relates mostly to sexual desire and physical aspects of a relationship, intimacy is associated with close, intimate understanding and trust between partners, whereas the commitment factor pertains to involvement in a relationship and commitment to the partner. In the context of the previously discussed functions and positive consequences of affectionate touch in close relationships, it may be presumed that affectionate touch strongly depends on the experience of love and all of its components: both as an expression of this feeling, and a way to nurture love by loving partners. It is important to note here that love is viewed as crucial for romantic relationships not only by scholars, who passionately debate about the functions of love^30,31^, but also by laypeople, whose immense interest in love is expressed in myriad songs, movies, and books^32,33^, across diverse cultures^34,35^. Love was found to be one of the top priorities in mate selection for both men and women across 37 countries—suggesting that it may be a culturally invariant predictor of relationship satisfaction^36^. This extends to motivations driving the stability of already existing partnerships^37^ and marriages^38^, wherein love is referred to as the most important factor associated with relationship satisfaction and partner commitment. Still, whether love is indeed related to affectionate touch remains yet to be examined.

Here, we explored the association of love and affectionate touch behaviors in romantic partnerships in two studies comprising a total of 7880 participants. In the first study, we used a cross-cultural survey conducted in 37 countries to test *whether love in romantic partnerships was universally associated with affectionate touch behaviors.* Our data included measurements of affectionate touch behaviors and love, and guided by previous findings in the area our research also comprised several individual- and culture-level predictors possibly affecting our outcomes. We selected factors used in our previous studies^6,39^ that were shown to relate to either love [i.e., having children^40,41^, relationship duration^29^] or touch [i.e., age^42,43^, gender, conservatism, interpersonal distance preferences^6^, socio-economic status (SES)^20^, and religiosity^6,44^]. For example, touch behaviors in close interpersonal relationships are typically used more by younger, female, and liberal people^6^. In the second study, using a more fine-tuned affectionate touch behavior measurement, we tested *whether the frequency of affectionate touch behaviors was related to love* and we further assessed the effect of potential moderator variables on that predicted association.

## Study 1

The primary aim of Study 1 was (a) to determine whether individual-level differences in love (i.e., an individual’s total score on the Triangular Love Scale) relate to an individual’s degree of affectionate touch to their romantic partner (i.e., the extent to which they hugged, kissed, touched and stroked their partner) and (b) to assess whether this relationship is potentially moderated by other, relevant factors. To achieve this aim, we re-analyzed a large international dataset on affectionate touch, love and potential moderating variables used in previous studies^6,39^.

## Materials and methods

### Participants

We retrieved data from all individuals who completed both the Sternberg’s Triangular Love Scale^29,39^ and the Affective Touch Questionnaire^6^ in the previous Global Survey, and who further declared having a romantic partner and having met up in person (physically) with this partner during the data collection period. The final sample of participants fulfilling these criteria was comprised of 7681 individuals from 37 countries aged between 15 and 87 years of age (*M* = 31.07, *SD* = 11.44; 0.9% minors) with 54.6% females. The relationship duration varied between less than one month and 50 years with a mean duration of 84 months (*SD* = 109 months), and 63.5% of the participants reported to have no children. See Table 1 below for statistics of sample sizes per country and country-level mean Affectionate Touch Variability Indices [DV]).

**Table 1:** Study 1 country-level descriptive statistics.

Country	Affectionate Touch Variability Index			
	*N*	*M*	*SD*	Cronbach’s α
Algeria	248	78.0	36.8	.910
Australia	245	95.3	16.7	.807
Austria	112	97.3	10.3	.510
Belgium	277	91.5	17.3	.549
Brazil	139	90.3	23.6	.820
Chile	113	80.3	23.3	.545
China	231	52.8	47.3	.961
Colombia	106	91.7	22.8	.845
Costa Rica	99	90.4	24.4	.849
Croatia	224	90.5	23.4	.812
Cuba	168	95.8	14.9	.736
El Salvador	51	79.4	25.8	.702
Estonia	148	82.6	20.3	.560
Georgia	123	84.8	28.4	.809
Germany	71	96.8	14.0	.806
Greece	131	88.2	25.1	.779
Hungary	804	92.6	19.5	.740
India	188	79.5	27.8	.678
Italy	268	89.6	21.8	.696
Lithuania	169	86.4	27.9	.829
Mexico	89	91.3	17.7	.614
Pakistan	314	61.1	37.3	.763
Peru	106	79.7	20.4	.617
Poland	378	90.4	23.3	.812
Portugal	166	89.6	22.0	.718
Romania	145	67.6	18.7	.456
Russia	155	86.3	28.2	.839
Serbia	364	87.8	22.5	.666
Slovakia	264	86.9	26.0	.793
Slovenia	467	91.5	21.1	.756
South Korea	130	68.5	37.9	.848
Spain	247	91.2	19.4	.632
Sweden	194	93.9	17.6	.731
The Netherlands	60	57.5	46.8	.956
Turkey	507	84.5	28.6	.801
Ukraine	102	58.6	31.0	.796
United states	78	91.3	24.5	.890
Total	7681	85.4	27.6	.792

### Procedure

A detailed description of the Global Survey data collection procedure can be found in other publications of our cross-cultural research group^6,39,45,46^. In brief, researchers in respective countries were asked to recruit adult male and female participants to take part in a large, cross-cultural study comprising several research questions. The participants who agreed to take part in the study completed paper-and-pencil questionnaires on a range of characteristics and behaviors, translated to their native language by the local collaborators. The samples in each country were to be as heterogeneous as possible, with a considerable proportion of community sample representatives and a proportion of students not exceeding 50%. All researchers followed the ethical guidelines of their countries and the study complied with the 1964 Helsinki Declaration as well as the American Psychological Association’s (APA) Ethical Principles of Psychologists and Code of Conduct. Each lab received ethical approval from their local Institutional Review Board (IRB) or gained approval through the Principal Investigator’s IRB (Institute of Psychology, University of Wrocław Ethics Committee). All subjects provided informed consent to be included in the study and informed consent was additionally obtained from a parent or a legal guardian of minors.

### Measures

#### Partner affectionate touch variability index

Participants were presented with four pictograms visualizing an embrace, stroke, kiss or hug and were surveyed whether they have performed this kind of touch with their romantic partner in the preceding week (see^6^ for details). Mirroring our previous cross-cultural study on affectionate interpersonal touch, we computed an individual-level Partner Affectionate Touch Variability Index for each participant, operationalized as the percentage of available touch behaviors used to one’s partner. The index reflects affective touch richness and has a higher resolution of possible values per person (0%, 25%, 50%, 75%, 100%, depending on how many of the four presented touch types participants reported) than a Prevalence Score (0% vs. 100%). In addition, Affectionate Touch Variability is less prone to ceiling effects than Touch Prevalence Score^6^.

#### Love

Love was operationalized following Sternberg’s Triangular Love Theory^29^ and measured with the use of Sternberg’s Triangular Love Scale (STLS^29^) in an appropriate language adaptation^39^. The STLS comprises three subscales, one per passion, intimacy and commitment components; there are a total of 45 items, 15 per subscale. The participants were asked to think about their romantic partner, and to indicate their agreement with each item using a 1 to 9 Likert-type scale. For the purpose of further analyses, we computed a single, mean Love score for each participant.

#### Additional constructs

As mentioned above, our analyses also included several additional variables which were previously shown to be linked to love or affectionate touch, namely gender, age, socio-economic status (SES), religiosity, preferred interpersonal distance, parental status, conservatism and relationship duration.

*Gender* was surveyed as male/female (dummy-coded as 0/1, respectively), and *age* as a numerical number in years. Our *SES* index was to show participants’ relative socio-economic situation in their country and it was operationalized as a mean value of two questions with response options provided on an eleven-point scale: (1) Please assess your economic situation in comparison to an average peer in your country (from 0—much worse than my average peer in my country to 10—much better than my average peer in my country); (2) Please assess how difficult it is for your family to meet the monthly payments (from 0—very difficult to 10—not difficult at all). *Religiosity* was measured with a single question “Are you religious?” with two response options (yes/no), dummy-coded as 1 and 0 for the purpose of further analyses. Mean *Preferred Interpersonal Distance* was assessed with a pictorial task^6^ in which the participants were asked to indicate how close they might get to various interaction partners while still feeling comfortable. The potential responses could range from 0 to 220 cm. Participants also declared whether they had *children* (yes/no) and their responses were further dummy-coded as 1 (parent) and 0 (childless). *Conservatism* was measured using Henningham’s Social Conservatism Scale^47^. *Relationship duration* was measured in months, and in the course of further analyses, we decided against the inclusion of relationship duration in the model, as it was highly correlated with age in our sample (*r* = 0.8).

### Data analysis

The primary aim of Study 1 was to determine whether individual-level differences in Love significantly predicted the Partner Affectionate Touch Variability Index across participating samples*.* To test the internal consistency of the Partner Affectionate Touch Variability Index, we computed Cronbach’s Alphas across the whole sample (α = 0.792) as well as for each single county (see Table 1). Thereafter, we tested how the Partner Affectionate Touch Variability Index was related to Love and to each of the subscales using Spearman correlation and we tested the difference between the correlation coefficients^48^. As the subscales of Love correlated highly with each other, subsequent analyses were based on the total Love scale.

To account for nested data, we used the multilevel linear model approach. The individual-level factors included in the model, in addition to Love, were Age, Gender, SES, Parental Status, Conservatism, Religiosity and Preferred Interpersonal Distance. Similarly, SES, Religiosity, and Conservatism could also be aggregated at the country level—and as such were included as country-level predictors of Partner Affectionate Touch Variability Index. All Individual-Level predictor variables were Group-mean centered, and all Cultural-Level predictor variables were Grand-mean centered. To ensure data validity, countries with less than 30 eligible individual cases were excluded from the analyses^49^. An unstructured covariance matrix was used in the Random-Effects Models and Scaled Identity in the earlier models, as these offered the best fit, based on the 2-log Likelihood criterion.

First, an Empty (or null) Model was run, to determine if Partner Affectionate Touch Variability Index differed across countries. Second, we computed a model with Love as a predictor of Partner Affectionate Touch Variability Index. Third, we added an Individual-Level Model with all fixed effects for all remaining Individual-Level predictor variables. Fourth, we added all Cultural-Level predictor variables into the model, and fifth, Love was added as a random slope in the final model to investigate if the relationship between Love and Partner Affectionate Touch Variability Index varied across countries. We tested how much variance in our dependent measure was accounted for in each model by computing Intraclass Correlations (ICCs) and by further comparing them between models (see Table 2). All data are available at *Love and Touch Studies*^50^ and statistical analyses were performed using SPSS v. 25. This study’s design and its analysis were not pre-registered.

**Table 2:** Multilevel analysis of the partner affective touch variability index.

Variables	Model 1: empty model	Model 2: individual model (Love only)	Model 3: complete individual model	Model 4: cultural model	Model 5: random slopes model
	Model estimates (Standard Error)				
Fixed intercept	84.13 (1.88)**	86.90 (1.56)**	87.72 (1.42)**	86.72 (1.24)**	86.77 (1.25)**
Individual-level variables					
Love		0.10 (.01)**	0.11 (.01)**	0.11 (.01)**	0.10 (.01)**
Age			− 0.34 (.04)**	− 0.34 (.04)**	− 0.34 (.04)**
Gender			− 0.93 (.64)	− 0.94 (.64)	− 0.92 (.64)
SES			0.29 (.17)	0.29 (.17)	0.27 (.17)
Children			0.74 (1.03)	0.76 (1.04)	0.78 (1.03)
Conservatism			− 0.71 (.18)**	− 0.71 (.18)**	− 0.68 (.18)*
Religiousness			− 0.39 (.32)	− 0.39 (.32)	− 0.36 (.32)
Interpersonal Distance			− 0.02 (.01)*	− 0.02 (.01)*	− 0.03 (.01)*
*Cultural level predictors*					
SES Country				− 2.76 (1.95)	− 2.58 (1.95)
Religiousness Country				− 6.53 (3.31)	− 6.53 (3.32)
Conservatism country				− 1.82 (1.36)	− 1.51 (1.37)
Random effects					
Inercept (Country) variance	125.47 (30.39)**	83.33 (20.60)**	66.77 (17.44)**	46.47 (12.83)**	47.56 (13.14)**
Slope (Love) variance					− .06 (.10)
Covariance between slope and Intercept					.002 (.001)*
Residual variance					
Residual variance	650.48 (10.52)**	522.71 (9.32)**	457.06 (9.51)**	456.89 (9.51)**	452.28 (9.46) **
Country variance	125.47 (30.39)	83.33 (20.60)	66.77 (17.44)**	46.47 (12.83)**	47.56 (13.14)*
Intra-class correlation (ICC)^a^	16.16%	13.75%	12.75%	9.23%	–
Added variance		14.91%	7.28%	27.61%	

^a^ICC = Country Variance/(Residual + Country Variance) × 100.

*p < *0.05.*

**p < *0.001.*

### Results

Love and each of the subscales were significantly related to the Partner Affectionate Touch Variability Index (Love Total: *R* = 0.193, Intimacy: *R* = 0.212; Passion: *R* = 0.180, Commitment: *R* = 0.158; each *p* < 0.001). The differences between correlation coefficients were significant for Intimacy vs Commitment (*p* = 0.003), but not for Intimacy versus Passion (*p* = 0.072) or Passion vs Commitment (*p* = 0.291). The descriptive statistics of all participants are presented in Table 1, whereas Table 2 presents the results of all computed models.

1. *Model 1: Empty Model* (i.e., no predictor variables included). This first model showed that there was a significant variation in the Partner Affectionate Touch Variability Index between countries. The Intraclass Correlation (ICC) indicated that 16.17 percent of the total variance in the Partner Affectionate Touch Variability Index was found between countries (See Table 2).
2. *Model 2: Love as a predictor* of the Partner Affectionate Touch Variability Index**.** Love was a significant and positive predictor of Affectionate Touch, suggesting that individuals expressing greater love to their romantic partners had a higher Partner Affectionate Touch Variability Index, i.e., they used more types of affectionate touch in their romantic relationships. As compared to model 1 (Empty Model), Love as a predictor accounted for an additional 14.96 percent of the variance in the Partner Affectionate Touch Variability Index (See Table 2).
3. *Model 3: Individual-Level Model.* After an inclusion of all additional, relevant individual-level predictors, Love continued to be significantly and positively related to the Partner Affectionate Touch Variability Index. Age, Conservatism and Preferred Interpersonal Distance also significantly and negatively predicted variation in the Partner Affectionate Touch Variability Index. Thus, higher variability in displays of affectionate touch toward partners were associated with younger age, lower conservatism levels, and preference for reduced interpersonal distance—as well as with greater love in a relationship. The inclusion of these variables explained an additional 7.27% of the variance in the Partner Affectionate Touch Variability Index as compared to the model comprising Love only.
4. *Model 4: Cultural-Level Model***.** While all cultural-level variables were negatively related to the Partner Affectionate Touch Variability Index*,* none reached statistical significance. Nonetheless, as these cultural-level variables improved the model fit (see Table 3), we decided to proceed with the model and found that it explained an additional 27.61% percent of variance in the Partner Affectionate Touch Variability Index*,* as compared to the model 3.
5. *Model 5: Random coefficients model.* The relationship between Love and the Partner Affectionate Touch Variability Index varied across countries, as indicated by a significant random slope of Love. There was also a significant variation around the intercept (suggesting that there was some variation across countries in the range of the Partner Affectionate Touch Variability Index). A visual inspection of scatter plots suggested that the slopes were more positive in some countries than others, but it should be noted that there were no notable negative slopes in our sample. Similarly, bivariate correlations between Love and the Partner Affectionate Touch Variability Index indicated that the correlations were marginally negative (i.e., < − 0.03) only in 3 countries, and this was likely due to a ceiling effect (they were observed in countries with very high intercept values of the Partner Affectionate Touch Variability Index*;* See Supplementary Fig. S1).

**Table 3:** Model fit statistics.

	Model 1: empty model	Model 2: individual model (Love only)	Model 3: complete individual model	Model 4: cultural model	Model 5: random slopes model
Deviance	71,683.780	57,678.846	41,819.762	41,806.188	41,790.398
Number of Parameters	3	4	11	14	16
Model comparison test*		Χ^2^ = 14,004.93	χ^2^ = 15,859.08	χ^2^ = 13.574	χ^2^ = 15.79
		df = 1	df = 7	df = 3	df = 2
		*p* < .0001	*p* < .0001	*p* = .004	*p* = 0.0004

*NB: Compared to the previous model.

## Brief discussion

Individuals expressing greater love toward their partners used more types of affectionate touch in their romantic relationships. Love was associated with affectionate touch behaviors in romantic partnerships across our large cross-cultural sample and the love factor added to our models accounted for 14.9% of the variance in the Partner Affectionate Touch Variability Index. Although other, theoretically relevant factors (age, conservatism, and preferred interpersonal distance) also moderated affectionate touch behaviors, it should be highlighted that the relationship between love and affectionate touch remained stable and positive across all computed models. Nevertheless, we note that the interpretation of our cross-cultural data is somewhat limited by the form of the affectionate touch survey (a few simple yes/no questions) and resulting distribution/ceiling effect problems, since affectionate touch was generally very diverse in romantic relationships.

## Study 2

Inspired by the outcomes of Study 1, we decided to further explore the association between love and touch behaviors by using a finer method of the affectionate touch measurement, namely touch frequency assessment, and by exploring the reliability of affectionate touch frequency reports. We invited a culturally homogenous community sample from Poland to complete an online survey that involved an assessment of the number of affectionate touch behaviors they performed toward their partners on a given day, and the Sternberg’s Triangular Love Scale.

## Materials and methods

### Participants

The participants for this study were a community sample recruited through social media announcements, institutional website, invitations distributed in a local shopping malls and schools, and through personal contacts of the researchers. The minimum sample size for a regression model with six predictors was estimated as 146 individuals [1 − β = 0.95; α = 0.05; medium effect size (*f* = 0.15) according to Cohen’s criteria^51^]. The recruited sample comprised 199 individuals (mean age = 30.20 ± 11.71; 29% men). All of them declared being in a relationship and having met up in person (physically) with their romantic partner on the day of the survey. The largest group consisted of married people (38%), followed by cohabiting (31%), dating (19%), individuals engaged to be married (19%), and participants who chose ‘other’ type of relationship as the best term to describe their relationship status (3%). Six participants were homosexual, 12 were bisexual and 181 were heterosexual. The mean duration of reported relationships was 8.98 (*SD* = 9.34) years and most participants were childless (67%). All participants were invited to participate in a follow-up survey, which included the touch questionnaire only, during the week following the first survey. Ninety people decided to complete the follow-up survey. The study was approved by the IRB and all participants provided written, informed consent prior to the study inclusion.

### Procedure

The study comprised two surveys that were to be completed online on separate days. The first survey contained demographic questions as well as the STLS^29^ and a modified Affective Touch Survey^6^, while the second survey contained only the Affective Touch Survey to enable a test–retest reliability analysis. Data across surveys were related using individual and anonymized identification codes. The surveys were created in the native language of the participants.

### Measures

#### Partner affectionate touch frequency index

Following Study 1, we defined affectionate touch behaviors as embrace, stroke, kiss and hug and the participants completed a simple questionnaire illustrated with graphics depicting each type of affectionate touch^6^. However, in this study, the subjects were asked to assess *how many times* they performed each type of touch toward their partner on the given day. The summarized frequencies of all affectionate touch types were afterwards divided by a total number of hours the person spent with a partner on the day of the study to obtain a single Partner Affectionate Touch Frequency Index [per hour] for each participant. The participants additionally reported whether they completed the survey on a working day or on their day off.

#### Love

Mirroring Study 1, Love was measured with Sternberg’s Triangular Love Scale^29^ in a relevant language adaptation^39^. We computed a total mean Love score, and for the purpose of further exploratory analyses we computed also separate mean values for Passion, Intimacy and Commitment subscales.

#### Additional constructs

Following the analytic approach presented in Study 1, the analysis of Love and Partner Affectionate Touch Frequency Index included several theoretically relevant factors, which were measured as reported in Study 1, namely: *Gender, Age, SES, Religiosity, Parental Status, Relationship Duration* and *Preferred Interpersonal Distance*. Due to an oversight in the study protocol, we did not include conservatism in the questionnaire.

### Statistical analyses

**Figure 1:**
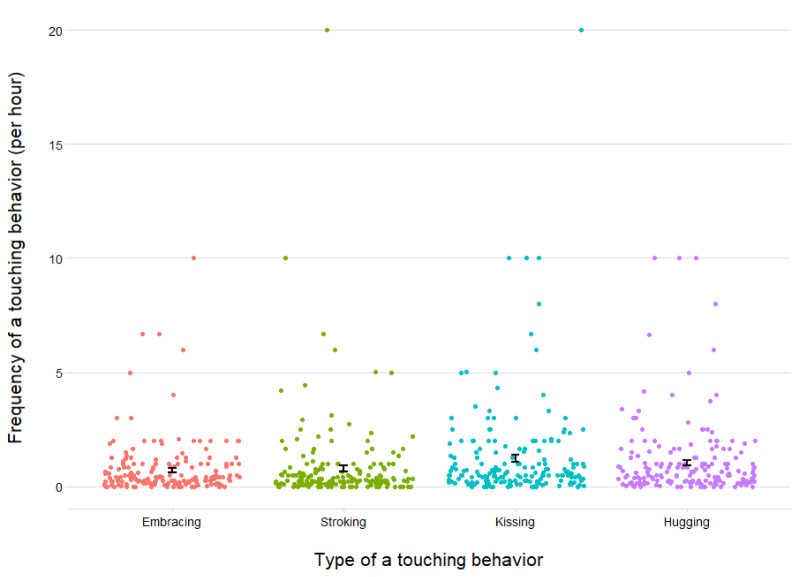
Partner Affectionate Touch Frequency across Four Types of Touch Behaviors (i.e., Embracing, Stroking, Kissing, and Hugging One’s Partner). *Note* Error bars represent standard errors.

We first used independent samples t-tests to test whether there were differences in the Partner Affectionate Touch Frequency Index regarding the day of the week (working day vs day off). As we found no significant differences between the two types of a day (*t*_*(193)*_ = 0.509, *p* = 0.611, *d* = 0.078), we did not include the type of the testing day in the further analysis (Fig. 1). Next, we calculated Mahalanobis Distance and relied on the usually recommended cutoff (i.e., < 0.001^52,53^) when screening for potential outliers–we excluded data from four participants based on this criterion.

In the second step, we checked for the normality of the distribution of all measurements and log-transformed the Partner Affectionate Touch Frequency Index as it violated the assumption of normality. Further, we checked for the reliability of the Partner Affectionate Touch Frequency measure, STLS, as well as Preferred Interpersonal Distance and self-assessed SES questions with Cronbach’s alphas, and we additionally examined intra-class correlation coefficients (ICC) for the Partner Affectionate Touch Frequency Index using data from individuals who completed the follow-up survey. We found all the applied scales to be acceptably reliable. The test–retest ICC for the Affectionate Touch questions was 0.770, Cronbach’s alpha for the Partner Affectionate Touch Frequency equaled 0.664, for STLS = 0.978, for Interpersonal Distance Preferences = 0.923, and for SES = 0.763. Finally, we again decided against an inclusion of relationship duration in the consecutive model, as it was extremely correlated with age in our sample (*r* = 0.9).

We then regressed the frequency of affectionate touch toward a partner on the participants’ Love (mean score of the STLS), Gender (with men dummy-coded as 0), Age, Religiosity (with non-religious participants coded as 0), Preferred Interpersonal Distance, SES, and Having Children (not having children coded as 0), and compared the models’ fit (F likelihood inclusion criterion = 0.05, exclusion criterion = 0.10). All data are available at *Love and Touch Studies*^50^ and statistical analyses were performed in R (4.1.0). The full list of used R packages along with references can be found in the Supplementary File S1. This study’s design and its analysis were not pre-registered.

### Results

Supplementary Table S1 presents participants’ descriptive data, and supplementary Table S2 reports correlations between the variables of interest. Overall, the total Love score was significantly correlated to Partner Affectionate Touch Frequency Index (*r* = 0.195, *p* < 0.01; see Table S1).

#### Partner affectionate touch frequency

The average frequency of touching behaviors per hour toward a partner were: 0.61 (*SD* = 0.91) for embracing, 0.67 (*SD* = 1.64) for stroking, 1.08 (*SD* = 1.90) for kissing, 0.89 (*SD* = 1.27) for hugging, altogether: 3.26 (*SD* = 4.11), see Fig. 1.

**Figure 2:**
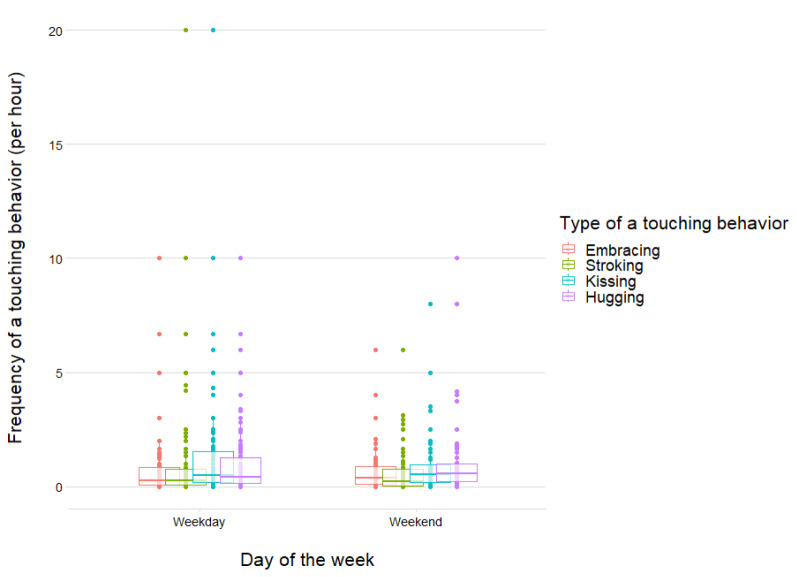
Partner Affectionate Touch Frequency on Weekdays and Weekends. *Note* Boxplots represent interquartile range of given Partner Affectionate Touch Frequency indices.

The variation of reported affectionate touch behaviors per hour was rather high, ranging from 0 to 10 hugs, 0 to 20 kisses and strokes, and 0 to 6.67 embraces. As mentioned before, Partner Affectionate Touch Frequency Index per hour did not differ between weekends and weekdays (compare Fig. 2).

#### Regression analysis

Love was found to significantly contribute to the Partner Affectionate Touch Frequency Index, regardless of the inclusion of other theoretically relevant factors. The Partner Affectionate Touch Frequency Index was also predicted by age and religiosity of the partners, and the overall model explained a significant proportion of variance in the data. The results of the linear regression analysis are shown in Table 4.

**Table 4:** A summary of the linear regression results with the partner affectionate touch frequency index as an outcome variable.

*Predictor*	Adj. *R*^2^ = 0.155, *F*_(7,182)_ = 5.934, *p* < 0.001			
	*β*	*95% CI*	*SE*	*p*
Love	0.164	[0.027, 0.302]	0.070	0.020*
Gender (0—Men, 1—Women)	− 0.109	[− 0.244, 0.026]	0.068	0.113
Age	− 0.235	[− 0.447, − 0.024]	0.107	0.029*
Religiosity (0—No, 1—Yes)	− 0.186	[− 0.327, − 0.046]	0.071	0.010*
Interpersonal distance preferences	− 0.125	[− 0.259, 0.009]	0.068	0.068
SES	0.022	[− 0.111, 0.155]	0.067	0.742
Children (0—No, 1—Yes)	− 0.009	[− 0.225, 0.207]	0.109	0.933

*p < 0.05.

We also explored the potential links between the Partner Affectionate Touch Frequency Index and each of the three components of love analyzed separately (i.e., Intimacy, Passion, and Commitment). Similar to Study 1, we observed high intercorrelations between the three components of love (i.e., Intimacy, Passion, and Commitment, see Table S2), and therefore we did not include them in one model. However, for exploratory purposes, we created three additional, separate models for each of the variables introduced as a predictor instead of a total love score. The patterns of results for passion and intimacy were very similar to the outcomes of the main analysis, while commitment was found not to be a significant predictor of Partner Affectionate Touch Frequency Index. The detailed results of these models can be found in the Supplementary Materials (Table S3).

## Brief discussion

Study 2 was conducted to assess the relationship between love and affectionate touch behaviors measured with a more fine-tuned scale than the measurement applied in Study 1. Passionate and intimate love significantly and positively predicted affectionate touch behaviors and this result was replicated regardless of the inclusion of other relevant factors in the analysis. Partners touched each other approximately 3 times per hour and the most frequently reported touch type was stroking. Further, our Affectionate Touch Frequency scale was characterized with satisfactory retest-reliability and the number of affectionate touch behaviors per hour did not depend on the day of the measurement (working day vs. weekend). Altogether, these data suggest that affectionate touch is a relatively stable characteristic of romantic relationships that is strongly and reliably predicted by passionate and intimate love to a partner.

## General discussion

Love was significantly related to affectionate touch behaviors in romantic partnerships both in our large, cross-cultural sample tested with a simple version of the touch scale, and in a second study involving a finer method to quantify the amount of affectionate touch. Crucially, the level of love was significantly and positively related to the variability of touch behaviors across a sample of participants from 37 countries, as well as in a culturally homogenous sample that reported the frequency of affectionate touch behaviors. Although touch has long been shown to be an important aspect of loving romantic relationships, this is the first comprehensive, cross-cultural research which indicates the crucial importance of love for affectionate touch behaviors and—conversely—of affectionate touch for nurturing love.

Although the direct association of love and affectionate touch may seem intuitive, ours is one of the few scientific studies that directly showed this relationship using empirical evidence. Almost 30 years ago, Dainton, Stafford and Canary^28^ showed a significant correlation between love and "physical affection displays" (e.g., hugging/kissing/cuddling, kissing goodbye, performing touch without sexual intent) as well as with the "satisfaction with physical affection". More recently, Jakubiak and collaborators^26^ additionally demonstrated that relationship quality was positively related to desire for touch in couples. Our research extends the conclusions of these works by showing that affectionate touch was reliably associated with love in a wide range of cultures from all over the world. None of the culture-level factors we included contributed significantly to the computed models. Although in some countries the observed, statistical relationships were weaker, this was probably driven by the extremely high touch variability reported by partners in these cultures, and related ceiling effects. Notably, the overall relationship of love and touch across the included samples was positive, and so it can be assumed that the value and meaning of touch for love in romantic relationships and the needs that affectionate touch behaviors may satisfy in loving couples are similar across cultures.

Our study provides novel insight to research on both love and affectionate touch. Mounting evidence suggests that experiencing love relates to psychological welfare, as indicated by increased well-being^54–56^, and higher happiness^57^. Love and attachment can further generate physiological benefits, such as better health^58^, or higher resilience against stress^59^ and pain^60^. We suggest that some of these beneficial effects of love can be mediated by the higher frequency of affectionate touch expressed in loving couples, as demonstrated in our study. This notion is additionally supported by numerous studies showing various physiological^61^ and psychological benefits of touch in a romantic partnership context^62^. These positive effects are additionally independent of other important relationship-related variables, such as attachment style^63^, and particularly salient in couples that report high relationship quality^64^ and satisfaction^11^.

Conversely, the Affection Exchange Theory^22^ proposes that both given and received affectionate messages enhance relational bonds. Therefore, apart from being a way to express love, desire, intimacy, etc., affectionate touch likely nurtures mutual affection, similar to other types of affectionate communication. The amount of received (but not expressed) affection in established romantic relationships was negatively related to perceptions of relational transgressions (i.e., severity, thoughts of rumination, and feelings of hurt) in a study by Horan^65^. Furthermore, Horan and Booth-Butterfield^23^ showed an additional association of received affection with relational satisfaction. In a similar vein, a 13-year study of married couples found that the degree of affectionate communication discriminated between divorced and stably married couples^66^. These works assessed touch as just one of several types of affectionate communication [e.g., “sharing physical affection” was only one of 15 items used by Huston^66^], but it is justified to assume that affectionate touch is not only a consequence of love to a partner, but that it is also one of the elements that an individual can use to enhance overall relationship stability and attachment, or promote love, as suggested by our study.

In Study 1 all touch subscales correlated with reported touch variability, but in Study 2, comprising a more detailed touch behaviors scale, we observed significant associations of love with touch for passion and intimacy, but not for a commitment love component^29^. Passion is the most physical of the three love components, while intimacy strongly connects with psychological closeness and mutual attachment^29^. Therefore, a consistent association of touch with these two love components as well as their predictive value for affectionate touch are not surprising. Touch conveys crucial and reliable information about the toucher’s feelings and intentions toward the receiver^62^ and even imagining a partner’s touch can be pleasant and sexually arousing^4^. In the case of the passion and intimacy components, touch behaviors seem to be a direct expression of the touching partner's needs for physical and psychological closeness. This closeness may, in turn, increase as a result of pleasant and nurturing touch experiences. Our findings shed some additional light on a previously reported higher frequency of touch in developing relationships^67^. These are likely the passionate and intimate love for the partner (particularly increased during the first phases of love^29^) that drive the need for more touch behaviors, and conversely—more frequent affectionate touch likely enhances these types of love. Interestingly, the predictive value of commitment was found not to be significant in Study 2. Again, referring to the original Triangular Love Theory^29^, we may assume that this is because commitment is the least “physical” among the love components and could be more related with other types of affection displays / partner retention strategies.

In our study, affectionate touch in romantic partnerships was predicted by several factors, namely age, interpersonal distance preferences and conservatism in Study 1 as well as age and religiosity in Study 2. Again, these findings suggest an important connection between religiosity, conservatism and more formalized (less freely and diversely expressed) affectionate behaviors, even in private, intimate relationships^6^. As mentioned in our previous work, the existing literature in this area is rather scarce, but the social environments characterized by high levels of these variables seem to shape more physically restrained expression of affection^68,69^. Interestingly, although both conservatism and religiosity promote norm adherence^70^ as well as significantly and negatively contribute to affectionate touch behaviors in partnerships, the effect of love on touch remained positive regardless of the inclusion of these factors in our models.

Our studies, or variance unexplained by our models, indicate a need to further pursue the research on love and affectionate touch in association with other individual- and culture-level factors. Touch avoidance, or a lower preference for interpersonal touch^18^, could be an important factor driving differences in affectionate touch in romantic partnerships. An attitude towards touch is highly individual, and touch does not have to be perceived as pleasant (e.g., in social anxiety^71^). Some people prefer avoiding touch or react to touch in a negative manner^19,72^, even when this type of affectionate behavior occurs in romantic relationships^26^. However, we should note that even for individuals less open to touch (such as people characterized by attachment avoidance) more touch in a romantic relationship can promote well-being^63^. Overall, it seems recommendable to include measures of the significance, meaning and/or preference of touch for each participant as moderating variables on both individual and cultural levels to future cross-cultural studies, as large variation in the population may further drive significant culture-level effects.

We observed minor differences in the moderating factors of touch in Study 1 as compared to our previous publication^6^ which used the same dataset. In particular, we have currently found no significant effect of country level variables, and the individual, preferred interpersonal distance was not a significant predictor of touch behaviors in a romantic relationship in the previous, cross-cultural analysis^6^. This is likely because variations in affectionate touch can be additionally explained by love (not included in the past publication), and the models in the current study focused on (fewer) variables, that were relevant to study also in the context of love. Further, many participants from the Global Survey completed surveys on either affectionate touch or love, and therefore the participating sample was less extensive in the current research (both in terms of size and the number of included countries). It seems worthy to continue the research on love and affectionate touch in association with other variables to clarify such inconsistencies. The scope of our research could also be extended by controlling other factors relevant in the context of touch perception, such as attachment patterns^73,74^, or by performing a more detailed measurement of constructs we included in our work (e.g., measuring a degree of religiosity rather than asking a yes/no question). We would also recommend a more extensive investigation of touch assessments. Although we found satisfactory test–retest reliability of our questionnaire, the method could be additionally validated in further studies, as the sample participating in our retest assessment was not very large and culturally homogenous. The touch survey could be supported by observational techniques to additionally verify the strength of observed associations. The link between affectionate touch and love could further be examined in longitudinal studies to test whether higher frequency of touching contributes to more intense feelings of love (as observed, e.g., for eye gaze^75,76^), especially because pleasantness of touch in close relationships seems to depend on continuous exposure to touch^77^.

In summary, touch is an extremely prevalent behavior in romantic relationships^6^ and people need more touch from a romantic partner than from other interaction partners^2^. Our research shows that one of the variables seemingly associated with differences in affectionate touch displays in romantic relationships is love. Regardless of the day of the week and the potentially confounding influence of other theoretically relevant factors included in our two studies, expression of affectionate touch was a relatively stable characteristic of romantic relationships that was reliably predicted by love for the partner. Interestingly, affectionate touch diversity was positively associated with all love components across our large, cross-cultural sample, while in the follow-up study touch frequency correlated with passion, intimacy, but not with the commitment love component.

## Supplementary Information

Supplementary Information.

## Data Availability

All data are published on-line (and referred to as Love and Touch Studies^50^) and the studies’ design and analyses were not pre-registered. The datasets used and/or analyzed during the current study are also available from the corresponding author on reasonable request.
