# Supplementary material for: Love and affectionate touch toward romantic partners all over the world
Source: Sci Rep. 2023 Apr 4;13:5497. doi: 10.1038/s41598-023-31502-1 (PMC10073073; doi:10.1038/s41598-023-31502-1)
Supplement: Supplementary file 1 — Supplementary Information. [file 41598_2023_31502_MOESM1_ESM.docx]

**Love and affectionate touch toward romantic partners all over the world**

**Supplementary Materials**

**Figure S1**

*Relationship between Love and Affectionate Touch Variability Index across countries in Study 1*


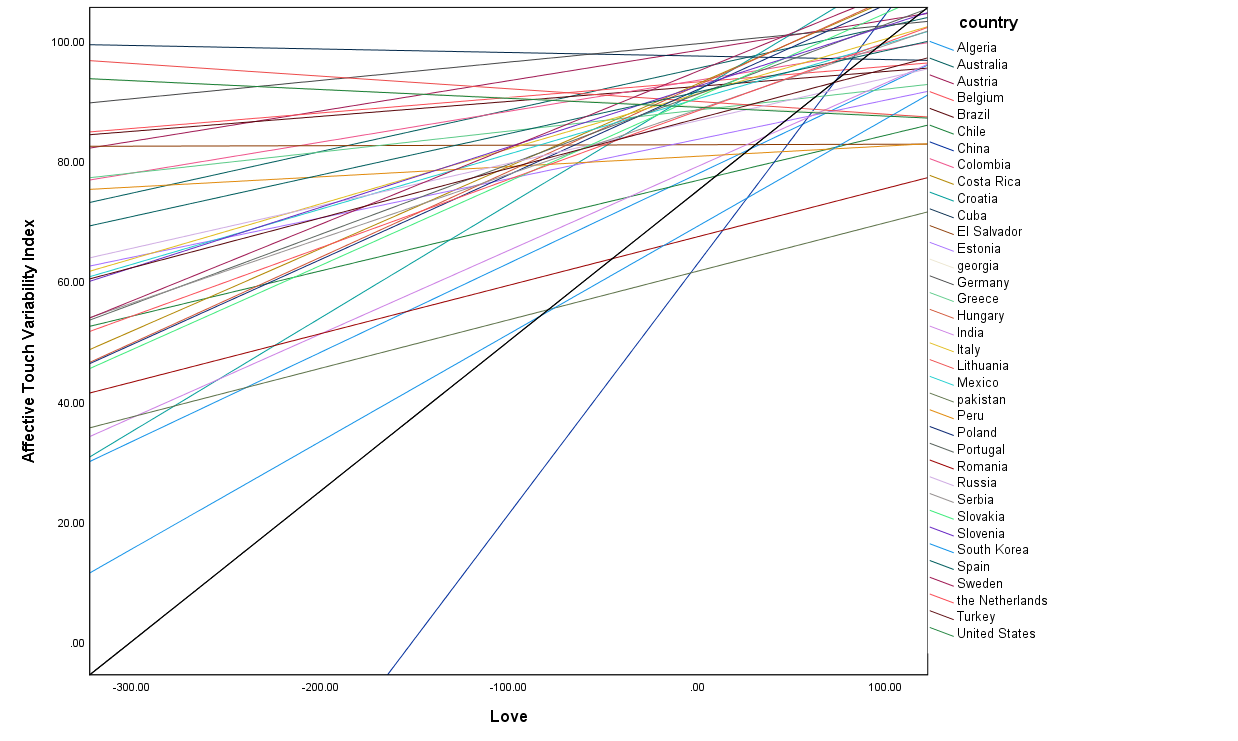


**Table S1**

*Descriptive data of participants from Study 2*

|  | ***N* (%) / Mean (*SD*)** | **Range** |
| --- | --- | --- |
| Men | 58 (30%) | - |
| Women | 137 (70%) | - |
| Age | 30.39 (11.75) | 18-59 |
| Affectionate touch ^a^ | 3.26 (4.11) | 0-24.44 |
| STLS ^b^ | 7.79 (1.13) | 1-9 |
| Intimacy ^c^ | 8.05 (1.06) | 1-9 |
| Passion ^c^ | 7.24 (1.39) | 1-9 |
| Commitment ^c^ | 8.11 (1.21) | 1-9 |
| SES ^d^ | 5.14 (1.27) | 2.33-7.67 |
| Interpersonal distance preferences ^e^ | 70.39 (39.87) | 0-190 |
| Religious individuals | 117 (60%) | - |
| Individuals with children | 66 (34%) | - |

*Note*. ^a^ - Affectionate touch to a partner frequency, ^b^ - Sternberg's Triangular Love Scale, ^c^ - A subscale of STLS, ^d^ - Self-assessed socio-economic status, ^e^ - Averaged scores of the preferred distance to a close female friend (or relative), a close male friend (or relative), a female acquaintance, a male acquaintance, a female stranger, and a male stranger.

**Table S2**

*Pearson Correlations Between the Variables of Interest in Study 2*

|  | **(1)** | **(2)** | **(3)** | **(4)** | **(5)** | **(6)** | **(7)** | **(8)** | **(9)** | **(10)** | **(11)** |
| --- | --- | --- | --- | --- | --- | --- | --- | --- | --- | --- | --- |
| (1) Affectionate touch | — |  |  |  |  |  |  |  |  |  |  |
| (2) Total Love | 0.195 ** | — |  |  |  |  |  |  |  |  |  |
| (3) Intimacy | 0.176 * | 0.909 *** | — |  |  |  |  |  |  |  |  |
| (4) Passion | 0.226 ** | 0.914 *** | 0.709 *** | — |  |  |  |  |  |  |  |
| (5) Commitment | 0.120 | 0.945 *** | 0.846 *** | 0.782 *** | — |  |  |  |  |  |  |
| (6) Age | -0.331 *** | -0.142 * | -0.102 | -0.238 *** | -0.048 | — |  |  |  |  |  |
| (7) Relationship length | -0.249 *** | -0.152 * | -0.088 | -0.268 *** | -0.053 | 0.833 *** | — |  |  |  |  |
| (8) Gender (0 - Male, 1 - Female) | -0.154 * | -0.177 * | -0.109 | -0.197 ** | -0.166 * | 0.043 | 0.070 | — |  |  |  |
| (9) SES | 0.045 | 0.076 | 0.201 ** | -0.008 | 0.046 | -0.025 | -0.028 | 0.000 | — |  |  |
| (10) Preferred distance | -0.147 * | -0.039 | -0.039 | -0.044 | -0.038 | 0.129 | 0.096 | 0.025 | -0.059 | — |  |
| (11) Religiosity (0 - No, 1 - Yes) | -0.194 ** | 0.121 | 0.053 | 0.109 | 0.179 * | 0.178 * | 0.207 ** | -0.073 | 0.065 | -0.023 | — |
| (12) Children (0 - No, 1 - Yes) | -0.284 *** | -0.141 | -0.097 | -0.247 *** | -0.038 | 0.778 *** | 0.696 *** | 0.062 | 0.016 | 0.052 | 0.274 *** |

Note. * *p* < 0.05, ** *p* < 0.01, *** *p* < 0.001.

**Table S3**

*A Summary of the Linear Regression Results with the Affectionate Touch to a Partner Frequency as an Outcome Variable and: Intimacy (first model), Passion (second model), and Commitment (third model) as Predictor Variables (Study 2)*

|  | *1 - Intimacy* | | | |
| --- | --- | --- | --- | --- |
|  | *Adj. r^2^ = 0.149, F_(7,182)_ = 5.722, p < 0.001* | | | |
| *Predictor* | *β* | *95% CI* | *SE* | *p* |
| Love | 0.144 | [ 0.007, 0.281] | 0.069 | 0.039* |
| Gender (0 – Male, 1 – Female) | -0.121 | [-0.255, 0.013] | 0.068 | 0.077 |
| Age | -0.240 | [-0.452, -0.028] | 0.108 | 0.027* |
| Religiosity (0 – No, 1 – Yes) | -0.170 | [-0.310, -0.030] | 0.071 | 0.017* |
| Preferred distance | -0.125 | [-0.259, 0.010] | 0.068 | 0.069 |
| SES | 0.005 | [-0.131, 0.141] | 0.069 | 0.941 |
| Children (0 – No, 1 – Yes) | -0.018 | [-0.234, 0.199] | 0.110 | 0.873 |
|  | *2 - Passion* | | | |
|  | *Adj. r^2^ = 0.159, F_(7,182)_ = 6.084, p < 0.001* | | | |
| *Predictor* | *β* | *95% CI* | *SE* | *p* |
| Love | 0.178 | [ 0.036, 0.319] | 0.072 | 0.014* |
| Gender (0 – Male, 1 – Female) | -0.1 | [-0.235, 0.035] | 0.069 | 0.146 |
| Age | -0.229 | [-0.441, -0.017] | 0.107 | 0.034* |
| Religiosity (0 – No, 1 – Yes) | -0.186 | [-0.327, -0.044] | 0.072 | 0.010* |
| Preferred distance | -0.129 | [-0.263, 0.005] | 0.068 | 0.058 |
| SES | 0.035 | [-0.098, 0.168] | 0.067 | 0.601 |
| Children (0 – No, 1 – Yes) | 0.001 | [-0.216, 0.218] | 0.110 | 0.992 |
|  | *3 - Commitment* | | | |
|  | *Adj. r^2^ = 0.145, F_(7,182)_ = 5.543, p < 0.001* | | | |
| *Predictor* | *β* | *95% CI* | *SE* | *p* |
| Love | 0.120 | [-0.018, 0.258] | 0.070 | 0.088 |
| Gender (0 – Male, 1 – Female) | -0.113 | [-0.248, 0.023] | 0.069 | 0.103 |
| Age | -0.246 | [-0.459, -0.033] | 0.108 | 0.024* |
| Religiosity (0 – No, 1 – Yes) | -0.176 | [-0.319, -0.034] | 0.072 | 0.016* |
| Preferred distance | -0.129 | [-0.264, 0.006] | 0.068 | 0.062 |
| SES | 0.028 | [-0.106, 0.162] | 0.068 | 0.681 |
| Children (0 – No, 1 – Yes) | -0.026 | [-0.244, 0.191] | 0.110 | 0.810 |

**Supplementary File S1**

*A full list of R packages used in Study 2*

1. Packages:

- Cowplot ^1^
- Dplyr ^2^
- E1071 ^3^
- Extrafont ^4^
- Ggeffects ^5^
- Ggplot2 ^6^
- Ggpubr ^6^
- Gridextra ^7^
- Paletteer ^8^
- Plyr ^9^
- Pracma ^10^
- Skimr ^11^
- Tidyr ^12^

1. References:
   1. Claus, O.W. *cowplot: Streamlined Plot Theme i Plot Annotations for 'ggplot2'. R package version 1.1.1*. <https://CRAN.R-project.org/package=cowplot> (2020).
   2. Hadley, W., Romain, W., Lionel, H., & Kirill, M. *dplyr: A Grammar of Data Manipulation. R package version 1.0.7*. <https://CRAN.R-project.org/package=dplyr> (2021).
   3. Davi, M., Evgenia, D., Kurt, H., Andreas, W., & Friedrich, L. *e1071: Misc Functions of the Department of Statistics, Probability Theory Group (Formerly: E1071), TU Wien. R package version 1.7-9*. <https://CRAN.R-project.org/package=e1071> (2021).
   4. Winston, C. *extrafont: Tools for using fonts. R package version 0.17*. <https://CRAN.R-project.org/package=extrafont> (2014).
   5. Lüdecke, D. ggeffects: Tidy Data Frames of Marginal Effects from Regression Models. *J. Open Source Softw.* ***3***, 772. <https://doi.org/10.21105/joss.00772> (2018).
   6. Alboukadel, K. *ggpubr: 'ggplot2' Based Publication Ready Plots. R package version 0.4.0.* <https://CRAN.R-project.org/package=ggpubr> (2020).
   7. Baptiste, A. *gridExtra: Miscellaneous Functions for "Grid" Graphics. R package version 2.3.* <https://CRAN.R-project.org/package=gridExtra> (2017).
   8. Hvitfeldt E. *paletteer: Comprehensive Collection of Color Palettes. version 1.3.0.* <https://github.com/EmilHvitfeldt/paletteer> (2021).
   9. Wickham, H. The Split-Apply-Combine Strategy for Data Analysis. *J. Stat. Softw.* ***40***, 1-29 (2011). <https://doi.org/10.18637/jss.v040.i01>.
   10. Hans, W.B. *pracma: Practical Numerical Math Functions. R package version 2.3.6.* <https://CRAN.R-project.org/package=pracma> (2021*).*
   11. Elin, W., Quinn, M., McNamara, A., Arino de la Rubia, E., Zhu, H., & Ellis, S. *skimr: Compact i Flexible Summaries of Data. R package version 2.1.3.* <https://CRAN.R-project.org/package=skimr> (2021).
   12. Wickham, H., & Girlich, M. *tidyr: Tidy Messy Data. R package version 1.2.1,* <https://CRAN.R-project.org/package=tidyr> (2022).
